# Does COVID-19 Clinical Status Associate with Outcome Severity? An Unsupervised Machine Learning Approach for Knowledge Extraction

**DOI:** 10.3390/jpm11121380

**Published:** 2021-12-17

**Authors:** Eleni Karlafti, Athanasios Anagnostis, Evangelia Kotzakioulafi, Michaela Chrysanthi Vittoraki, Ariadni Eufraimidou, Kristine Kasarjyan, Katerina Eufraimidou, Georgia Dimitriadou, Chrisovalantis Kakanis, Michail Anthopoulos, Georgia Kaiafa, Christos Savopoulos, Triantafyllos Didangelos

**Affiliations:** 1 First Propaedeutic Department of Internal Medicine, Aristotle University of Thessaloniki, AHEPA University Hospital of Thessaloniki, 54621 Thessaloniki, Greece; evelinakotzak@hotmail.com (E.K.); mixavtt@hotmail.com (M.C.V.); ariadni22@yahoo.gr (A.E.); kristine.kasazjyan77@gmail.com (K.K.); katerina.effraim@gmail.com (K.E.); georgettin@gmail.com (G.D.); vaalkakanis@gmail.com (C.K.); mixalisonco@gmail.com (M.A.); gdkaiafa@yahoo.gr (G.K.); chrisavopoulos@gmail.com (C.S.); didang@auth.gr (T.D.); 2 Emergency Department, AHEPA University Hospital, Aristotle University of Thessaloniki, 54621 Thessaloniki, Greece; 3 Advanced Insights, Artificial Intelligence Solutions, Ipsilantou 10, Panorama, 55236 Thessaloniki, Greece; aanagn@advancedinsights.eu

**Keywords:** COVID-19, clinical severity, clustering, unsupervised machine learning, Gaussian mixture models

## Abstract

Since the beginning of the COVID-19 pandemic, 195 million people have been infected and 4.2 million have died from the disease or its side effects. Physicians, healthcare scientists and medical staff continuously try to deal with overloaded hospital admissions, while in parallel, they try to identify meaningful correlations between the severity of infected patients with their symptoms, comorbidities and biomarkers. Artificial intelligence (AI) and machine learning (ML) have been used recently in many areas related to COVID-19 healthcare. The main goal is to manage effectively the wide variety of issues related to COVID-19 and its consequences. The existing applications of ML to COVID-19 healthcare are based on supervised classifications which require a labeled training dataset, serving as reference point for learning, as well as predefined classes. However, the existing knowledge about COVID-19 and its consequences is still not solid and the points of common agreement among different scientific communities are still unclear. Therefore, this study aimed to follow an unsupervised clustering approach, where prior knowledge is not required (tabula rasa). More specifically, 268 hospitalized patients at the First Propaedeutic Department of Internal Medicine of AHEPA University Hospital of Thessaloniki were assessed in terms of 40 clinical variables (numerical and categorical), leading to a high-dimensionality dataset. Dimensionality reduction was performed by applying a principal component analysis (PCA) on the numerical part of the dataset and a multiple correspondence analysis (MCA) on the categorical part of the dataset. Then, the Bayesian information criterion (BIC) was applied to Gaussian mixture models (GMM) in order to identify the optimal number of clusters under which the best grouping of patients occurs. The proposed methodology identified four clusters of patients with similar clinical characteristics. The analysis revealed a cluster of asymptomatic patients that resulted in death at a rate of 23.8%. This striking result forces us to reconsider the relationship between the severity of COVID-19 clinical symptoms and the patient’s mortality.

## 1. Introduction

Disease outbreaks have overwhelmed humanity several times throughout world history. The latest one, a new type of coronavirus, SARS-CoV-2, appeared in China during the last months of 2019, and rapidly spread worldwide [1]. In February 2020, the World Health Organization (WHO) appointed the name COVID-19 to the disease that was caused from this kind of coronavirus, and in March 2020, declared COVID-19 a global pandemic [2]. Today, the explosion of the virus continues to spread and mutate continuously. As a result of the international concern, public health and all scientific interests focused on the global fight against the pandemic [3].

COVID-19 is a disease with multiple clinical manifestations of different severity and in a number of cases, with rapid development [4]. The spectrum of the symptoms includes asymptomatic patients, patients with mild symptoms and critically ill patients [5]. Hence, the development of the disease is not predictable, and the prompt identification of severe patient cases is of great importance, due to the chance of early intervention, especially in patients with lethal outcomes. In addition, the intervention aims to prevent the severity of the disease to go from mild to severe [4,6,7]. The above shows the importance of the patient triage for proper care, due to limited hospital resources. Moreover, the financial poverty of the health systems increased mortality during the COVID-19 pandemic [8].

Thus, the effective prognostic model systems for early determination of COVID-19 disease severity are essential [8]. The optimal models should include prognostic biomarkers that allow for the faster referral of the patients to targeted treatment, and hospitalization of the patients in early stages of the disease [9]. There are a few models that used a duple of clinical symptoms and laboratory parameters for the diagnosis of critically ill and fatal patients, but these failed because of their complexity, the pressing pandemic situations and the lack of expert knowledge and experience in management of the disease, in combination with the burnout that was observed in physicians [10,11].

In order to tackle the issues of this global novel pandemic, the World Health Organization (WHO), scientists and researchers in healthcare, are in search of novel tools that will assist them in COVID-19 infected patients’ screening [12], identifying optimal clinical trials [13] and monitoring and inhibiting the virus’ spread [14]. Machine learning (ML) and artificial intelligence (AI) have been identified in recent studies as two of the most promising technologies in general, but also specifically in healthcare [15]. ML and AI, alongside the rapid advancement in processing speeds and availability of memory, offer better speed-up, scale-up, reliability and in some cases better performance than human experts in specific tasks [16]. As a consequence, physicians are eager to collaborate with data scientists in order to tackle the challenges of this COVID-19 pandemic and its consequences on patients, healthcare providers and in health care systems, in order to develop solutions that will assist, but never replace, physicians and medical experts, alleviate human errors due to fatigue and provide support in decision making, especially in critical conditions [3,17,18,19,20,21,22].

AI and especially ML are the research areas that deal with innovative computational models, that possess the ability to learn from data without explicitly being told how to [23]. These models, when trained and applied carefully, have been proven to effectively assist during decision-making processes with various professionals, policymakers and stakeholders related to healthcare [24]. These models have the ability to adapt themselves by using learning algorithms, which perform iterative mathematical operations in order to extract knowledge or infer conclusions, only based on the underlying patterns of raw data [25].

Concerning the COVID-19 clinical diagnosis, such models have been tested on severely infected patients, with the aim to optimize their screening, minimize care delays, reduce their hospitalization time and diminish mortality [18,26]. All based on ML approaches, these studies aimed to identify comorbidities, laboratory results and demographic factors as important predictors for the classification of patients with high mortality due to COVID-19 [27,28]. The main issue of these studies, however, was the reliability of the models, which had low sensitivity in the classification results, mostly due to the small dataset size [11,29,30]. This outcome, together with the current and continuous research on targeted treatment based on specific biomarkers, which are still undefined [31], are deterrent factors to the application of such models in real-life conditions [8].

Nevertheless, ML and AI applications have been continuously studied in a variety of applications during this global pandemic, such as vaccine development, infection tracing and screening of patients [28]. Other applications focus on the crucial challenge of patient triage, resource allocation and Intensive Care Unit (ICU) prioritization [6,7]. The large variety of symptoms and their respective potential outcomes have proven that all methods proposed so far rely on complex parameter sets and intuitive systems for COVID-19 severity, based on the physician’s experience and intuition [11].

There is a dire need for categorizing COVID-19 infected patients based on the severity of their condition, since this is a physician’s priority when a patient is admitted [11]. Triage of COVID-19 infected patients is of the utmost importance in order to provide intensive management for the patients that are in high-risk of mortality [32]. Physicians, due to the uncertain behavior of the virus and their own physical and mental exhaustion, need an assistive tool for the triage of COVID-19 infected patients [33]. Such tools should be reliant on an accessible and easily acquired set of input variables, and at the same time, be intuitive, accessible and inexpensive. AI and ML algorithms have proven that they can offer valuable solutions to the assistance of medical personnel in the fight against this pandemic [34]. However, they have been used in a supervised manner, meaning that some sort of “answers” are inferred, or an arbitrary classification is taken into consideration [35].

Due to insufficient data for COVID-19 and inaccurate severity or mortality biomarkers, the triage of COVID-19 infected patients is challenging [36]. Therefore, there is an urgent need for assistive healthcare systems that offer support in tasks that up to now relied on expert knowledge only, i.e., trained physicians and medical staff. Moreover, fatigue can impede physicians’ and nurses’ performance, especially during the challenging times of the pandemic. The pressing situations configured by the pandemic increased not only the healthcare system’s burden, but a psychological burden as well [37]. During the pandemic, frontline medical staff have had direct contact with infected patients and expressed greater mental distress [38], while the psychological challenges of the pandemic have also been described by physicians [39,40]. The unprecedented strain the pandemic has caused on healthcare workers internationally [41] has translated to burnout among physicians [42]. The chronic occupational stress evolved into burnout, during the pandemic, as a result of physical and emotional exhaustion, quarantine life meters, depersonalization and decreased personal accomplishment [43,44]. Thus, the development of proven assistive AI methodologies and systems can only complement a physician’s work, by providing advanced insights, extracting knowledge and providing steady performance without compromises regardless of the external pressure or fatigue.

The aim of this study is to propose a methodology that infers clusters of COVID-19 infected patients with deep correlations and underlying common characteristics, and extracts knowledge based on their features. This methodology is developed in an unsupervised manner, utilizing machine learning algorithms, meaning that there is no prior classification system, correct “answers” or optimal input taken into consideration. To the authors’ knowledge, this is a novel attempt to solve the problem of unsupervised clustering of infected patients based on their biomarkers, symptoms and comorbidities, in order to correlate their severity. Related studies utilizing machine learning algorithms focus on supervised learning tasks such as classification, whereas the proposed methodology extracts knowledge from unlabeled data with a focus in creating “patient clusters”. This study does not adopt any triage or categorization of infection severity, since the causality between symptoms and outcomes is still not clear, and would additionally include a bias to the outcome. Such information offers added value to physicians, especially in decision-making situations, where the goal is to reduce the mortality of virus infected patients. Additionally, the design of the proposed study includes exclusively easily accessible input variables (vital signs and comorbidities of the patients) that have no financial cost, and hematological/biochemical tests that mainly are routine tests for the patients at the Emergency Department.

## 2. Materials and Methods

**Figure 1:**
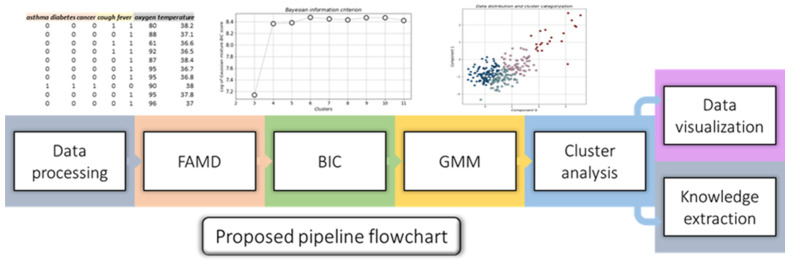
Flowchart of the proposed methodology.

An algorithmic pipeline based on unsupervised machine learning algorithms, which aims to operate in tandem with physicians and provide additional knowledge for the proper categorization of COVID-19 infected patients based on their severity, is proposed in this study. This methodology consists of 5 steps, as seen in the detailed flowchart in Figure 1.

Each component of the pipeline is further discussed in the following sections.

### 2.1. Data Processing

Data were collected and stored in separate Microsoft Excel files (.xlsx), which were loaded into memory. A script concatenated them all into a single data frame where they were checked for NaN (Not a Number) values. Because of the nature of the data, patients with missing information were discarded entirely from the dataset, since information inference would be a biased practice for this particular application. Next, we applied data normalization by scaling all numerical variables between the (0,1) range, so that the range of all numerical variables was the same, and any bias towards certain variables was avoided [45].

### 2.2. Factor Analysis of Mixed Data (FAMD)

The factor analysis of mixed data (FAMD) is a statistical exploratory method that handles both numerical and categorical data [46]. FAMD reduces the size of a dataset by applying a principal component analysis (PCA) on the numerical part of the dataset and a multiple correspondence analysis (MCA) on the categorical part of the dataset. Therefore, in cases where mixed types of variables are present in the dataset, FAMD offers the mechanism to identify the similarities between multiple data points, taking into consideration any variable type that is available in the dataset. This method offers a useful tool for dimensionality reduction that handles mixed types of data and can assist in data exploration as well as visualization.

### 2.3. Bayesian Information Criterion (BIC)

The Bayesian information criterion (BIC) is a statistical criterion that is based on the likelihood function [47]. BIC is usually used for model selection, when out of a finite set of models, differentiating by range of parameters, the model with the lowest BIC is the one fitting on the data the best way possible. The mathematical definition of BIC is given:

$$BIC=kln\left(n\right)-2ln\left(\hat{L}\right)$$

where $\hat{L}$ is the maximum value of the likelihood function, *n* is the sample size and *k* is the model’s estimated number of parameters.

### 2.4. Gaussian Mixture Models (GMM)

Gaussian mixture models (GMM) are probabilistic models aimed at the representation of normally distributed subpopulations within a total population [48]. It is an unsupervised learning method, meaning that knowledge about which subpopulation a data point belongs to is not required, as the model learns subpopulation automatically. GMM are generally used in cases where data seem to appear at more than one peak in their distribution (multimodal distribution). Because many simple distributions are unimodal (have one peak), a way to model a multimodal distribution is by assuming that this distribution is created by multiple unimodal distributions. As Gaussian distribution is the most common unimodal distribution, data with multimodal distribution can be modeled as a mixture of many Gaussian distributions. A GMM is effectively the weighted sum of M component Gaussian densities, as it can be seen in the following equation:

$$p\left(\vec{x}\right)=\overset{K}{\underset{i=1}{\sum }}\varphi_{i}N(\vec{x}|{\vec{\mu }}_{i},\Sigma_{i})$$

where $\vec{x}$ is a D-dimensional continuous-valued data vector (features), $\varphi_{i}$ are the mixture component weights and $N(\vec{x}|{\vec{\mu }}_{i},\Sigma_{i})$, with i=1,…,K are the component Gaussian densities. The mathematical formula of each component density is presented below:

$$N\left(\vec{x}|{\vec{\mu }}_{i},\Sigma_{i}\right)=\frac{1}{{\left(2\pi \right)}^{\frac{D}{2}}{\left|\Sigma_{i}\right|}^{\frac{1}{2}}}exp\left(-\frac{1}{2}{\left(\vec{x}-{\vec{\mu }}_{i}\right)}^{T}\Sigma_{i}^{-1}\left(\vec{x}-{\vec{\mu }}_{i}\right)\right)\;$$

where *D* represents the total dimensions of each data point, ${\vec{\mu }}_{i}$ is the mean value and $\Sigma_{i}$ the covariance.

### 2.5. Cluster Analysis

A cluster analysis consists of two parts, the knowledge extraction, where useful statistics are derived from the dataset based on the newly provided cluster information, and the data visualization, where data are plotted in 2D- or 3D-planes in order to create optical intuition to the problem. Both are equally important since the visualizations offer instant cognitive comprehension of the problem, and the extracted knowledge offers a more in-depth understanding since first- or even second-order statistics might be involved.

### 2.6. Use Case—Patients’ Information

A total of 268 patients have been included in this study, respecting the ethical committee guidelines by the Declaration of Helsinki. This study took place at University General Hospital of Thessaloniki AHEPA. The study protocol was approved by the Scientific Committee of the hospital (13/5/2021, #389) and is registered at clinicaltrials.gov (NCT05119465). A thorough and detailed data collection process was designed in order to collect information for the patients, without disturbing the clinical treatment, or upsetting them in the process. A detailed list of the used variables can be seen in Table 1.

Out of the total number of patients, one-hundred thirty-six (136) were male (50.75%) and one-hundred thirty-two (132) female (49.25%), leading to a balanced dataset, and removing any bias concerning sex. The mean value of the total number of the patients’ age was 59.34 years old. In total, thirty-eight (38) variables were included in the dataset, not including death and ICU hospitalization since they were considered outcomes, covering symptoms, comorbidities and measurements that were taken at admission. The percentages of the patients’ comorbidities for each coexisting condition were: 25.37% for cardiovascular disease, 2.98% for chronic kidney disease, 3.73% for chronic obstructive pulmonary disease, 1.87% for asthma, 17.91% for diabetes, 36.57% for arterial hypertension, 3.36% for immunosuppression and 5.60% for cancer. The percentages for each patient’s symptoms at admission were: 27.24% cough, 76.87% fever, 30.97% weakness, 1.87% headache, 4.10% dizziness, 1.49% abdominal pain, 2.24% nausea, 6.34% diarrhea, 3.73% vomit, 1.12% anosmia, 0.75% tastelessness and 1.87% throat pain. Counts and percentages of the number of patients suffering from some of the aforementioned comorbidities are presented in Table 2.

**Figure 2:**
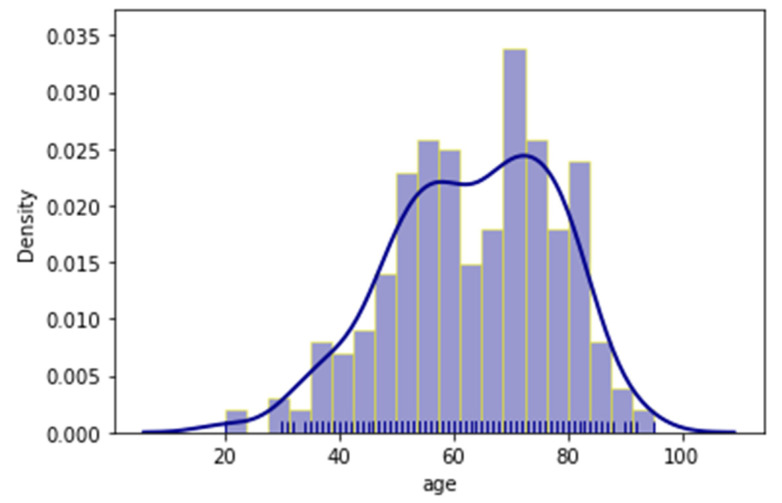
Distribution plot of the patients’ age.

**Figure 3:**
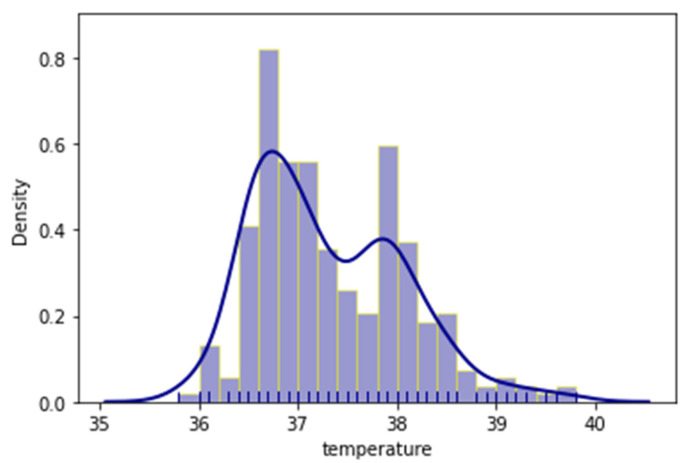
Distribution plot of the recorded temperatures.

**Figure 4:**
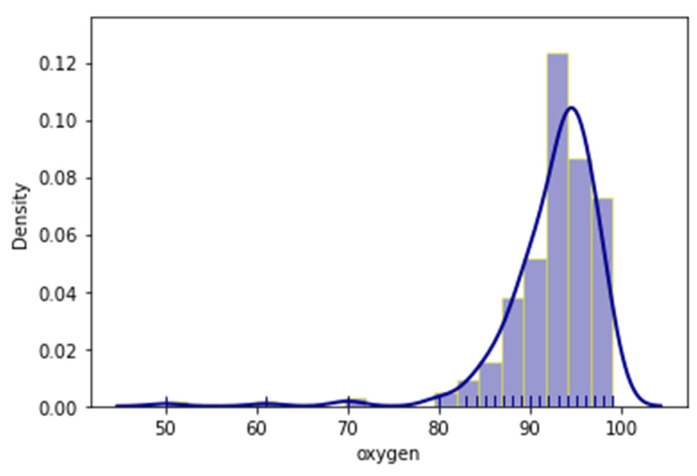
Distribution plot of the recorded oxygen saturation.

As mentioned before, the outcome of each patient’s admission was also documented with two (2) variables, whether the patient had been admitted in the ICU, or had a lethal outcome. Additionally, distribution plots of the age, temperature and oxygen variables were provided respectively in Figure 2, Figure 3 and Figure 4. Distribution plots assist with a better visual understanding and offer initial intuition on the dataset.

It is apparent that, even though the patients ranged from early twenties (20) to late nineties (90), a large number were just over fifty (50) and the majority were around seventy (70) years old. Regarding temperature, most patients had slightly higher temperatures than normal; however, there was a measurable spike in the 38 °C region. The oxygen measurements followed a more normal distribution with most patients having measurements of around 92.5% saturation.

## 3. Results

The proposed methodology was applied to the dataset as described in the previous section. The algorithm was entirely programmed in Python 3.8 with the use of Numpy and Pandas libraries for mathematical operations and table manipulation, Scikit Learn for data normalization and unsupervised clustering algorithms, Prince library for the principal component analysis and Matplotlib for visualization. All software requirements were under free usage licenses, and the hardware requirements were low. Specifically, each run of the entire pipeline, from data loading to the knowledge extraction and data visualization, would take approximately thirty (30) seconds in an Intel^®^ Core™ i7-8550U CPU at 1.80GHz.

**Figure 5:**
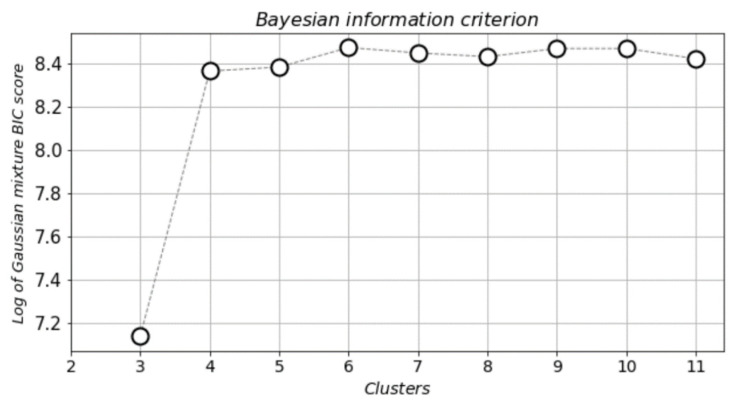
Cluster selection using Bayesian information criterion (BIC).

The computational pipeline began with the data loading and preparation, immediately followed by the FAMD method that aimed to reduce the number of variables and size of the dataset, with the minimal possible reduction in the contained information. Reducing the size of multidimensional data also benefits the visualization of clusters, which is an important part in a cluster analysis. The total number of input variables was thirty-eight (38), and the two (2) outcome variables were used solely for cross-referencing purposes. The application of FAMD was designed in such a way, so that the variables were reduced to a total of two (2) components. Next, the BIC was calculated using GMM in order to identify the number of clusters under which the best grouping of patients occurred. BIC was used to identify the desired number of clusters, in the present study’s case the minimum number, where the clusters differentiated in terms of features with each other significantly. The optimal number for the present study was found to be four (4) clusters. A smaller number would result in overlapping clusters and a higher number a further segmentation of clusters that offer no added knowledge. The results of the BIC analysis can be seen in Figure 5.

**Figure 6:**
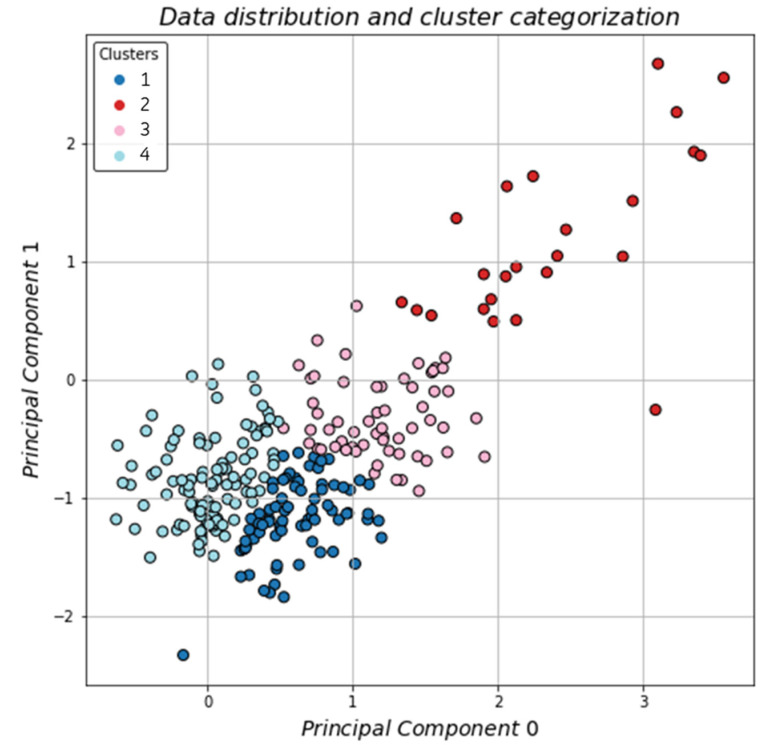
Clustering of the dataset on a 2D-plane.

The plot displays clearly that the optimal number of clusters was four (4), which was the selected and proposed number for the study. The GMM algorithm was applied on the dataset, and the results can be seen in Figure 6.

**Figure 7:**
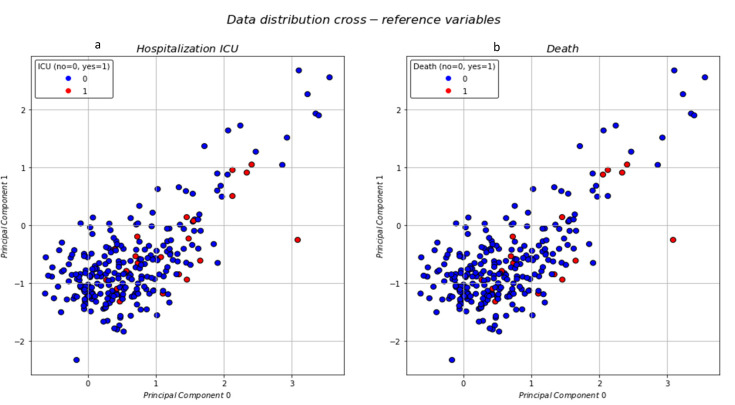
Data distribution with patient’s ICU admittance (**a**) and death (**b**).

The clusters were well defined with the most distinct area being the one on the top right of the plane with the sparse data, almost looking like outliers. The next step was to visualize the data distribution and identify if the reference variables were occurring within certain clusters. In Figure 7a, the patient’s admittance to the ICU was visualized and in Figure 7b the patient’s death.

The data distribution for both death and ICU hospitalization show that most occurrences could be found mainly in two clusters. The preliminary results, based solely on the visualized distributions, as seen in the plots, offer some intuition; however, the detailed statistical analysis is further discussed in the next section.

From our data, admittance to the ICU and mortality were highly correlated; however, they were not exclusive attributes of only one cluster. On the contrary, patients who had been hospitalized in the ICU or had died due to COVID-19 could be mainly found in two clusters. General statistics concerning each cluster can be found in Table 3.

From our data and the cluster analysis that is presented in Table 4, we extracted knowledge based on the categorical variables, as well as the reference variables that were used in this study.

## 4. Discussion

The application of artificial intelligence in all areas of medicine can offer a large variety of benefits and significant impacts [49]. However, since 2020 there has been a global pandemic due to the outbreak of COVID-19 and its consecutive mutations. At the moment of the study submission, a fourth wave of the pandemic is expected, without even considering viral mutations which would likely be immune to the produced vaccines, and could consecutively create additional pandemic waves. Physicians and medical staff are already worn out, and hospitals have faced large influxes of infected patients. One very serious issue is the triage of infected patients based on their severity and expected outcome.

The proposed methodology, based on unsupervised machine learning, is able to categorize patients with similar characteristics into clusters. Due to its distribution-based approach, the GMM algorithm is selected for the clustering, which is also used to identify the optimal number of clusters with the use of the BIC method. The created clusters are cross-referenced with two variables, the patient’s admission to the ICU and lethal outcome of the patient, in order to identify the value of the created clusters. Additionally, each cluster is studied individually in order to extract statistics and possibly identify the features that characterize each one.

It is apparent and well-known that admittance to the ICU and mortality are highly correlated, however, they are not exclusive attributes of only one cluster. On the contrary, patients who have been hospitalized in the ICU or have died due to COVID-19 can be mainly found in two clusters. General statistics concerning each cluster can be found in Table 3.

From Table 3, useful knowledge can be extracted on why the clusters were created this way by the AI algorithm. For example, the clusters have high variance when concerning oxygen and d-dimers; however, d-dimers have large dispersion which is apparent due to the large standard deviation. In the present study, the apparent oxygen levels can be scientifically explained, since COVID-19 is a type of respiratory disease [50,51]. The d-dimers’ high levels are associated with abnormal coagulation and are correlated with severe COVID-19 disease [52]. Ferritin, LDH and albumin have similar mean values in groups of two and small standard deviation which means that they appear as common characteristics in more than a single cluster. LDH serum levels are considered as an important biomarker for lung infection [53] and contemporary studies reveal the association with higher COVID-19 mortality [28]. COVID-19 disease is considered as a cytokine storm syndrome, which includes high levels of inflammatory markers [54,55]. Ferritin is a key biomarker of the immune system, with immunosuppressive and proinflammatory actions participating in a cytokine storm and is associated with COVID-19 mortality [8,56]. LDH is a marker of cellular injury and is associated with respiratory function [57]. The latest studies reveal that high levels of LDH are related with greater COVID-19 disease severity [58].

Other variables have standard deviations that are very large; therefore, it is not practical to extract conclusions from them. A noteworthy result is that the temperature has highly similar mean values and small standard deviations across all clusters; therefore, it might not be affecting the patient’s categorization in the degree that was previously thought [59]. In addition, the present study design indicates the temperature has been recorded only once, upon patient admission at the Emergency Department of the hospital. The above could be considered as a limitation of the study.

The cluster analysis in Table 4 displays extracted knowledge based on the categorical variables, as well as the reference variables that are used in this study. Cluster 2 has by far the highest percentage (23.8%) in ICU admitted patients, as well as in mortality. What is also noteworthy about cluster 2 is that the majority of symptoms is at 0%, or the lowest percentage compared to the other clusters, except cough, which is significantly high (33.3%). This cluster of patients also have higher levels of WBC, PLT, AST, ALT, CRP and IL6 and lower levels of Ht and eosinophils, compared to other clusters. Moreover, cluster 2 has no significant comorbidities and in fact 0% immunosuppression. The most noteworthy fact for cluster 2 is that the symptoms that lead the patients to the physician are mild. Hence, there is a novel discovery stemming from the deep underlying connection found with the proposed approach, that patients infected by COVID-19, with no or mild symptoms could have severe disease and high mortality risk. This conclusion is considered a stepping stone for further investigation in future studies.

On the other hand, cluster 1 has the lowest percentages in ICU admission (1.8%) and death (0.9%) even though there is a measurable percentage for most recorded symptoms. Patients in cluster 1 appear with lower concentrations of WBC, CRP, IL6 and higher levels of Ht, compared to other clusters. Clusters 3 and 4 are more alike, with minor differences in total; however, the ICU admission and death in cluster 3 are higher than in cluster 4, even though various symptoms or comorbidities appear less frequently.

Medical decisions for the patient management are based, not only on hematological/biochemical tests, but mainly on the clinical status of the patient. Moreover, the physician’s clinical practice prescribes personalized decision-making. Thus, patients that belong in cluster 2, the cluster with a higher need for ICU hospitalization and mortality, since they appear with no or mild symptoms, would not normally alert physicians to the need for more intensive management. Consequently, the present study unveils novel and useful knowledge for the COVID-19 pandemic management.

## 5. Conclusions

The present study introduces the use of clustering for the categorization of COVID-19 infected clinical patients. A novel methodology is proposed, based on unsupervised machine learning techniques, namely Gaussian mixture models, the factor analysis of mixed data and Bayesian information criterion, for the creation of clusters containing patients that have similar characteristics. The motivation for this approach is two-fold, firstly to extract new knowledge that correlates clinical variables and comorbidities with symptoms and outcomes, and secondly to create an assistive system that will be able to work in tandem with the physicians.

Regarding the knowledge extraction, the proposed approach identified a cluster of patients who, even though they displayed no or mild symptoms, had a high rate of morbidity (23.8%). This high rate had an added weight, especially when comparing with the other clusters, in which the patients had significantly lower morbidity rates (0.9–8.6%), even though the recorded symptoms were high. This result poses high significance since the correlations and causations between the input biomarkers and the clinical output is still under heavy investigation for COVID-19.

Concerning the application of such a methodology in real-life conditions, it is implied that it would operate exclusively as an assistive system, which would support decision-making from physicians. Such decision support systems have become increasingly important in all domains, and will become even more so in the future, especially in healthcare. The reason why there is increasing trust in said systems stems from the fact that AI and ML have increased their effectiveness and potential so much through the last years and proven that their application can be mutually beneficial for both the providing, as well as the receiving end, i.e., medical staff and patients.

Future plans include the clinical validation of the results that have arisen from the present study and expansion of the dataset with newly collected data from patients. Unfortunately, COVID-19 is still an active and deadly pandemic, and scientists are expecting the rise of the fourth wave, along with the domination of the Delta variation of the mutated virus, which has a significant impact even on vaccinated individuals. Therefore, the next steps will focus on the extraction of valuable new knowledge, as well as the application of the proposed methodology in real-life conditions for assistive purposes.

## Figures and Tables

**Table 1:** The variables contained in the dataset.

Type	Variable
General (mixed)	sex, age
Comorbidities (categorical)	Cardiovascular_disease, chronic_kidney_disease, chronic_obstructive_pulmonary_disease, asthma, diabetes mellitus, arterial_hypertension, immunosuppression, cancer
Symptoms (categorical)	cough, fever, weakness, headache, dizziness, abdominal_pain, nausea, diarrhea, vomit, anosmia, tastelessness, throat_pain
Measurable (numerical)	oxygen, temperature, d-dimmers, WBC, Ht, eosinophils, basophils, PLT, ferritin, AST, ALT, LDH, albumin, CRP, IL6, lymphocytes
Reference (mixed)	Hospitalization_ICU, death

**Table 2:** Count and percentages of the number of patients with present comorbidities.

Comorbidities	Counts (*n*)	Percentages (%)
Cardiovascular Disease	68	25.37
Chronic Kidney Disease	8	2.98
Chronic obstructive pulmonary disease	10	3.73
Asthma	5	1.87
Diabetes mellitus	48	17.91
Arterial Hypertension	98	36.57
Immunosuppresion	9	3.36
Cancer	15	5.60

**Table 3:** Mean values and standard deviations of each cluster’s numerical variables.

Cluster #	1	2	3	4
total patients	110	21	58	79
sex (male/female)	29/81	13/8	41/17	53/26
age	61.3 ± 15.0	65.4 ± 13.9	64.5 ± 13.9	65.2 ± 13.9
oxygen	94.2 ± 3.3	87.5 ± 11.4	90.6 ± 6.1	92.9 ± 3.1
temperature	37.1 ± 0.7	37.5 ± 0.8	37.3 ± 0.8	37.5 ± 0.7
d-dimers	451.2 ± 697.2	1833.7 ± 3845.4	1020.7 ± 1233.4	483.4 ± 539.9
WBC	6.2 ± 2.8	11.3 ± 5.8	10.4 ± 8.3	6.5 ± 2.8
Ht	36.3 ± 10.9	29.2 ± 16.9	32.9 ± 15.9	31.1 ± 17.0
eosinophils	0.08 ± 0.4	0.008 ± 0.01	0.04 ± 0.1	0.1 ± 0.7
basophils	0.04 ± 0.1	0.02 ± 0.02	0.02 ± 0.04	0.03 ± 0.07
PLT	217.2 ± 92.2	291.3 ± 136.3	240.9 ± 134.5	194.4 ± 80.7
ferritin	291.2 ± 217.7	2084.4 ± 2353.3	811.7 ± 720.8	421.4 ± 280.4
AST	29.7 ± 14.3	146.3 ± 103.8	58.5 ± 33.2	30.6 ± 13.6
ALT	25.4 ± 15.5	112.3 ± 91.2	47.1 ± 38.7	23.1 ± 12.9
LDH	266.8 ± 83.0	646.9 ± 250.8	473.6 ± 38.7	306.7 ± 89.6
albumin	3.6 ± 0.4	3.3 ± 0.4	9.7 ± 48.4	8.3 ± 41.9
CRP	4.0 ± 4.0	179.6 ± 747.2	9.4 ± 9.2	11.9 ± 47.3
IL6	28.2 ± 32.6	160.9 ± 246.6	94.9 ± 144.7	31.4 ± 23.6
lymphocytes	2.39 ± 8.6	1.3 ± 0.8	18.1 ± 129.3	1.2 ± 0.6

**Table 4:** Ratio of positive over total occurrences of each cluster’s categorical variables.

Cluster #	1	2	3	4
total patients	110	21	58	79
sex (male/female)	29/81	13/8	41/17	53/26
cardiovascular disease (%)	21 (19.1%)	7 (33.3%)	12 (20.7%)	28 (35.4%)
chronic kidney disease (%)	1 (0.9%)	1 (4.8%)	3 (5.2%)	3 (3.8%)
chronic obstructive pulmonary disease (%)	3 (2.7%)	1 (4.8%)	3 (5.2%)	3 (3.8%)
asthma (%)	3 (2.7%)	1 (4.8%)	1 (1.7%)	0 (0%)
diabetes (%)	13 (11.8%)	3 (14.3%)	8 (13.8%)	24 (30.4%)
arterial hypertension (%)	28 (25.5%)	4 (19.0%)	25 (43.1%)	41 (51.9%)
immunosuppression (%)	3 (2.7%)	0 (0%)	1 (1.7%)	5 (6.3%)
cancer (%)	6 (5.5%)	2 (9.5%)	2 (3.4%)	5 (6.3%)
cough (%)	19 (17.3%)	7 (33.3%)	19 (32.8%)	28 (35.4%)
fever (%)	63 (57.3%)	4 (19.0%)	49 (84.5%)	77 (97.5%)
weakness (%)	23 (20.9%)	2 (9.5%)	20 (34.5%)	38 (48.1%)
headache (%)	1 (0.9%)	0 (0%)	1 (1.7%)	3 (3.8%)
dizziness (%)	6 (5.5%)	0 (0%)	2 (3.4%)	3 (3.8%)
abdominal ache (%)	2 (1.8%)	0 (0%)	1 (1.7%)	1 (1.3%)
nausea (%)	2 (1.8%)	0 (0%)	0 (0%)	4 (5.1%)
diarrhea (%)	7 (6.4%)	0 (0%)	4 (6.9%)	6 (7.6%)
vomit (%)	4 (3.6%)	0 (0%)	3 (5.2%)	3 (3.8%)
anosmia (%)	2 (1.8%)	0 (0%)	0 (0%)	1 (1.3%)
tastelessness (%)	1 (0.9%)	0 (0%)	0 (0%)	1 (1.3%)
throat ache (%)	4 (3.6%)	0 (0%)	0 (0%)	1 (1.3%)
hospitalization ICU (%)	2 (1.8%)	5 (23.8%)	8 (13.8%)	6 (7.6%)
death (%)	1 (0.9%)	5 (23.8%)	5 (8.6%)	6 (7.6%)
